# Supplementary material for: Bacterial polyphosphates induce CXCL4 and synergize with complement anaphylatoxin C5a in lung injury
Source: Front Immunol. 2022 Nov 3;13:980733. doi: 10.3389/fimmu.2022.980733 (PMC9669059; doi:10.3389/fimmu.2022.980733)
Supplement: Supplementary file 1 [file DataSheet_1.docx]

**Fc-PPX1 Protein Sequence:**

Protein Length=635 MW=71833.0 Predicted pI=5.94

1 MEPKSCDKTH TCPPCPAPEL LGGPSVFLFP PKPKDTLMIS RTPEVTCVVV DVSHEDPEVK

61 FNWYVDGVEV HNAKTKPREE QYNSTYRVVS VLTVLHQDWL NGKEYKCKVS NKALPAPIEK

121 TISKAKGQPR EPQVYTLPPS RDELTKNQVS LTCLVKGFYP SDIAVEWESN GQPENNYKTT

181 PPVLDSDGSF FLYSKLTVDK SRWQQGNVFS CSVMHEALHN HYTQKSLSLS PGKDDDDKMS

241 PLRKTVPEFL AHLKSLPISK IASNDVLTIC VGNESADMDS IASAITYSYC QYIYNEGTYS

301 EEKKKGSFIV PIIDIPREDL SLRRDVMYVL EKLKIKEEEL FFIEDLKSLK QNVSQGTELN

361 SYLVDNNDTP KNLKNYIDNV VGIIDHHFDL QKHLDAEPRI VKVSGSCSSL VFNYWYEKLQ

421 GDREVVMNIA PLLMGAILID TSNMRRKVEE SDKLAIERCQ AVLSGAVNEV SAQGLEDSSE

481 FYKEIKSRKN DIKGFSVSDI LKKDYKQFNF QGKGHKGLEI GLSSIVKRMS WLFNEHGGEA

541 DFVNQCRRFQ AERGLDVLVL LTSWRKAGDS HRELVILGDS NVVRELIERV SDKLQLQLFG

601 GNLDGGVAMF KQLNVEATRK QVVPYLEEAY SNLEE

**Fc-PPX1-D127N Protein Sequence:**

Protein Length=635 MW=71844.7 Predicted pI=5.81

1 MEPKSCDKTH TCPPCPAPEL LGGPSVFLFP PKPKDTLMIS RTPEVTCVVV DVSHEDPEVK

61 FNWYVDGVEV HNAKTKPREE QYNSTYRVVS VLTVLHQDWL NGKEYKCKVS NKALPAPIEK

121 TISKAKGQPR EPQVYTLPPS RDELTKNQVS LTCLVKGFYP SDIAVEWESN GQPENNYKTT

181 PPVLDSDGSF FLYSKLTVDK SRWQQGNVFS CSVMHEALHN HYTQKSLSLS PGKDDDDKMS

241 PLRKTVPEFL AHLKSLPISK IASNDVLTIC VGNESADMDS IASAITYSYC QYIYNEGTYS

301 EEKKKGSFIV PIIDIPREDL SLRRDVMYVL EKLKIKEEEL FFIEDLKSLK QNVSQGTELN

361 SYLVNNNDTP KNLKNYIDNV VGIIDHHFDL QKHLDAEPRI VKVSGSCSSL VFNYWYEKLQ

421 GDREVVMNIA PLLMGAILID TSNMRRKVEE SDKLAIERCQ AVLSGAVNEV SAQGLEDSSE

481 FYKEIKSRKN DIKGFSVSDI LKKDYKQFNF QGKGHKGLEI GLSSIVKRMS WLFNEHGGEA

541 DFVNQCRRFQ AERGLDVLVL LTSWRKAGDS HRELVILGDS NVVRELIERV SDKLQLQLFG

601 GNLDGGVAMF KQLNVEATRK QVVPYLEEAY SNLEE
