# Supplementary material for: Bacterial polyphosphates induce CXCL4 and synergize with complement anaphylatoxin C5a in lung injury
Source: Front Immunol. 2022 Nov 3;13:980733. doi: 10.3389/fimmu.2022.980733 (PMC9669059; doi:10.3389/fimmu.2022.980733)
Supplement: Supplementary file 2 [file Image_1.pdf]

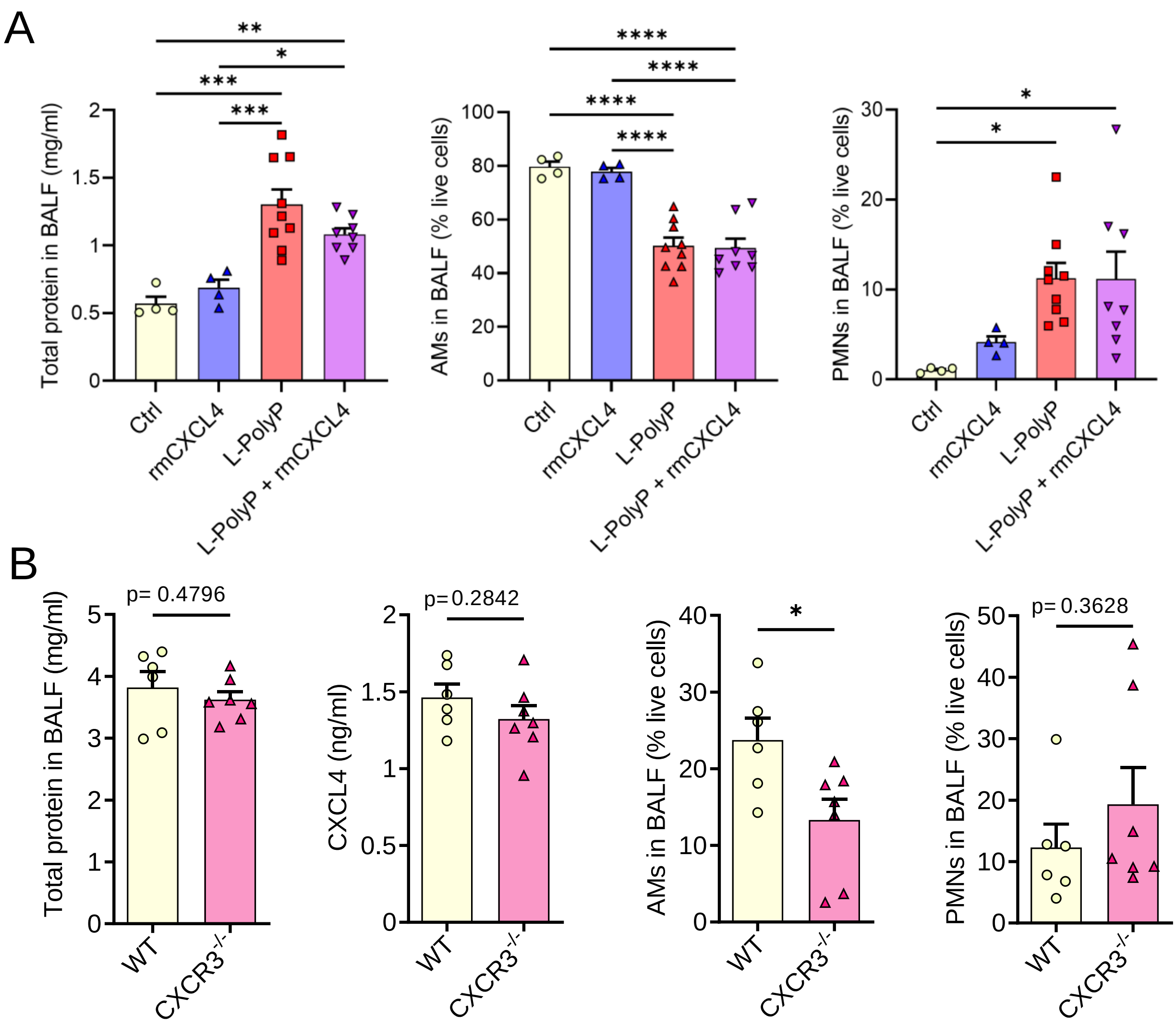

SUPPLEMENTARY FIGURE 1. Role of CXCL4 and CXCR3 in polyphosphate-induced lung injury. (**A**) C57BL/6J mice (WT) received recombinant mouse CXCL4 (rmCXCL4, 500 ng/mouse i.t., n=4), long-chain polyphosphates (40µl of 20 mM/mouse i.t., n=9) alone or in combination (n=8). The sham control mice (Ctrl) received buffer (40µl PBS i.t., n=4). Total protein and frequencies of live $CD11c^{+}SiglecF^{+}$ alveolar macrophages (AMs) and live $Ly6G^{+}$ polymorphonuclear neutrophils (PMNs) were quantified in BALF by BCA protein assay and flow cytometry, respectively, 8 h. (**B**) C57BL/6J and $CXCR3^{-/-}$ mice received long-chain polyphosphates (40µl of 10 mM/mouse i.t.). Total proteins, CXCL4 (ELISA), frequencies of live $CD11c^{+}SiglecF^{+}$ AMs, live $Ly6G^{+}$ PMNs were quantified in BALF (n=6-7/group), 12 h. Data are presented as mean ± SEM; *p < 0.05; **p < 0.01; ***p < 0.001; ***p < 0.0001.
