# Supplementary material for: Bacterial polyphosphates induce CXCL4 and synergize with complement anaphylatoxin C5a in lung injury
Source: Front Immunol. 2022 Nov 3;13:980733. doi: 10.3389/fimmu.2022.980733 (PMC9669059; doi:10.3389/fimmu.2022.980733)
Supplement: Supplementary file 3 [file Image_2.pdf]

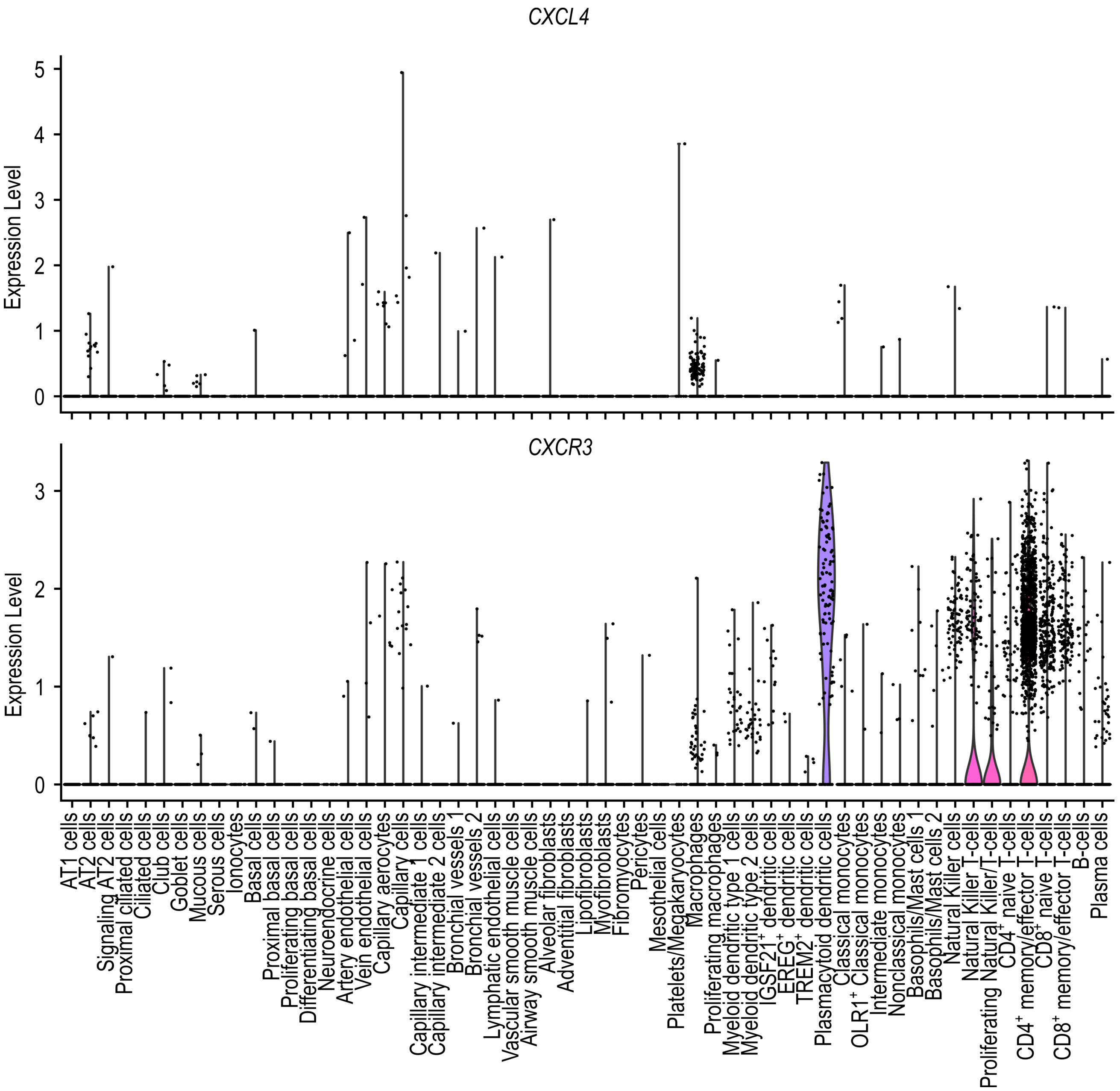

SUPPLEMENTARY FIGURE 2. Expression of *CXCL4* (*PF4*) and *CXCR3* in normal human lung transcriptomes. Violin plot showing expression levels from single cell RNA-sequencing data from adult human lungs (n=3). The data is based on scRNA-sequencing by Travaglini et al. from healthy, uninvolved lung tissues from patients (aged 46 years [male], 51 years [female], 75 years [male]) undergoing lobectomy for pulmonary tumors.
